# New Prognostic and Predictive Markers in Cancer Progression

**DOI:** 10.3390/ijms21228667

**Published:** 2020-11-17

**Authors:** Susan Costantini, Alfredo Budillon

**Affiliations:** Unità di Farmacologia Sperimentale - Laboratori di Mercogliano, Istituto Nazionale Tumori “Fondazione G. Pascale”—IRCCS, 80131 Napoli, Italy

Biomarkers are a critical medical need for oncologists to predict and detect disease and to determine the best course of action for cancer patient care. Prognostic markers are used to evaluate a patient’s outcome and cancer recurrence probability after initial interventions such as surgery or drug treatments and hence to select follow-up and further treatment strategies. On the other hand, predictive markers are increasingly being used to evaluate the probability of benefit from clinical intervention(s), driving personalized medicine. Evolving technologies and the increasing availability of “multi-omics” data are leading to the selection of numerous potential biomarkers based on DNA, RNA, miRNA, protein, and metabolic alterations within cancer cells or tumor microenvironments, which may be combined with clinical pathological data to greatly improve the prediction of both cancer progression and therapeutic treatment responses. Indeed, the search for new prognostic and predictive cancer biomarkers is the object of many studies performed on preclinical cancer models, as well as biofluids and tissue samples from cancer patients. However, few biomarkers have progressed in the last years from discovery to become validated tools to be used in clinical practice [1].

This Special Issue comprises eight review articles and five original studies on novel potential prognostic and predictive markers for different cancer types.

Among the reviews, two are focused on miRNAs that represent a family of small non-coding RNAs recently emerging as potential predictors of prognosis and/or anticancer drug efficacy, as well as novel anticancer targets. MiRNAs have been described as onco-suppressors or onco-genes, depending on the cellular context and their biological targets [2]. In recent years, many investigations have confirmed the presence of a stable form of circulating miRNAs in human body fluids, including blood, saliva, and urine [3]. In this context, Takeuchi et al. summarized the miRNAs known to be deregulated in head and neck squamous cell carcinoma (HNSCC), with a specific focus on laryngeal cancers, describing miRNas predicting initiation, progression, and prognosis; those associated withradio-, chemo-, and thermal- resistance; and also those correlated with infection (e.g., HPV) and life habit. Notably, the authors concluded that the simultaneous evaluation of more miRNAs regulating multiple target genes might have higher diagnostic, prognostic and therapeutic performance, as well as higher sensitivity than individual miRNA assays, because they better recapitulate the multistep process leading to cancer. On the other hand, Motti et al. provided an updated overview about miRNA deregulation in melanoma, suggesting their critical role as putative diagnostic and/or prognostic biomarkers in this disease. Moreover, since some miRNAs have been found to regulate the mitogen-activated protein kinase (MAPK) signaling pathway [4] or immune checkpoint expression [5], the authors discussed in more detail which miRNAs play an important role in melanoma cell resistance to MAPK and/or immune checkpoint inhibitors, evidencing the predictive potentialities of circulating miRNAs to detect and monitor melanoma responsiveness to targeted and immune therapies.

Anyhow, immune checkpoint inhibitors (ICI), beyond melanoma are used today in clinical practice to treat a large number of tumor types. However, ICI are effective in a small subgroup of cancer patients and, hence, a great effort has been dedicated to the identification and development of predictive biomarkers for ICI response [6]. In this regard, in a review article, Sabbatino et al. focused on the putative role of the human leukocyte antigen (HLA) system as a predictive biomarker for ICI-based cancer immunotherapy. The authors described: (i) the HLA system’s structure and function; (ii) HLA defects with their clinical significance in cancer patients, underlining the potential predictive role of the HLA as a biomarker of response to checkpoint-based immunotherapy in cancer patients; and (iii) the main molecules/drugs able to restore HLA function and usefulness to implement novel therapeutic strategies in cancer patients. Moreover, in a research paper, Baek et al. re-classified the urothelial cancer subtypes, focusing on ICI responsiveness. Several previous studies subdivided urothelial cancer patients in two immunotherapy-associated subgroups: the genomically unstable (GU) subtype of the Lund classification and the neuronal subtype in The Cancer Genome Atlas (TCGA) classification [7,8]; on this basis, the authors performed both hierarchical clustering and survival analysis using gene expression profiling in the IMvigor 210 cohort comprising 298 urothelial cancer patients, and evidenced that patients with upregulation of the cell cycle, DNA replication, and DNA damage and downregulation of the TGFβ and YAP/TAZ pathway were more responsive to ICI therapy. In another paper, Quagliariello et al. focused on Nod-like receptor (NLR) family pyrin domain containing 3 (NLRP3), a key player inimmune-related events involving viral and bacterial infection [9], suggesting that it could be a putative marker of cardiotoxicity induced by the ICI. In detail, with the aim of studying if hyperglycemia could affect ipilimumab-induced anticancer efficacy and toxicity, taking advantage of in vitro co-culture of human peripheral blood mononuclear cells (hPBMCs) with either cardiomyocytes or estrogen-responsive (MCF-7) and triple-negative (MDA-MB-231) breast cancer cells, the authors evaluated ipilimumab’s effect under different glucose concentrations. Interestingly, high levels of glucose during ipilimumab treatment increased cardiotoxicity and NRLP3 levels, and induced decreased cancer cell mortality; on the other hand, co-treatment with ipilimumab and empagliflozin, a sodium glucose co-transporter 2 inhibitor, under high glucose or shifting from high glucose to low glucose, was able to increase ipilimumab responsiveness and to decrease cardiotoxicity and NRLP3 levels.

Acetylation represents a post-translational modification regulating protein expression and function and it is regulated by acetyltransferases catalyzing the transfer of acetyl residues on proteins and by deacetylases that remove those residues.In this regard, in a review article, Kim et al. focused on N-α-acetyltransferase 10 (NAA10), targeting the *N*-terminal α-aminogroup of nascent proteins as well as internal protein lysine residues. The authors showed that NAA10 is an interesting player to regulate cell proliferation, differentiation, migration, autophagy, and apoptosis. They also highlighted NAA10 overexpression in different cancer types and correlation with overall survival rates and disease recurrence.

Several reviews and papers in this Special Issue reported data related to biomarkers of specific cancer types.

In a review article, Majewska et al. focused on head and neck paragangliomas (HNPGL) and, on the basis of PubMed and ScienceDirect databases, highlighted known genetic changes as well as epigenetic modifications associated with HNPGL, as well the potential practical applications of such alterations and also in the light of next-generation sequencing (NGS) technology. The authors analyzed both somatic and germline mutations, evidencing that, among others, succinate dehydrogenase complex iron sulfur subunit B (SDHB) mutations lead to metastasis development in 40% or more of patients. Overall, they concluded that fumarate hydratase (FH); the proto-oncogene tyrosine-protein kinase receptor RET; succinate dehydrogenase complex, subunits A, B, C, and D (SDHA, SDHB, SDHC, SDHD); and von Hippel–Lindau tumor suppressor (VHL) should be routinely determined in HNPGL patients in order to discover genetic syndromes and for correct prognostic evaluation.

Regarding prostate cancer, in a review article, Shorning et al. focused their attention on the PI3K–AKT–mTOR pathway, which is dysregulated in a high proportion of prostate cancer patients and is associated with castrationresistance [10]. The authors, by reviewing the genetic alterations leading to the activation of this pathway, discussed the interplay with androgen receptor (AR), MAPK, and WNT, which cooperate to promote prostate tumorigenesis and therapy-resistant disease progression, concluding that a clear knowledge of thePI3K–AKT–mTOR signaling network can be useful for improvingpatient stratification and to identify more targeted therapeutic approaches. In a research paper, Polo et al. applied some integrated and bioinformatics approaches to study the network of interactions between the 25 proteins belonging to the selenoprotein family, named selenoproteins, to identify the more correlated nodes (HUB nodes) and to analyze the correlation between selenoprotein gene expression and patient outcome in 10 solid tumors. To confirm some of the correlations suggested by the bioinformatics analyses, they evaluated the gene expression level of the 25 selenoproteins and of the HUB nodes identified in two androgen receptor-positive and two androgen receptor-negative prostate cancer cell lines, compared with normal human prostate epithelial cells by RT-qPCR analysis. In this way, the authors identified some selenoproteins, such as GPX2, MSRB1, SELENOK, SELENOI, and SELENOS, which are correlated with HUB nodes and are involved in prostate cancer, concluding that their combined evaluation could improve prostate cancer patient prognosis and outcome predictions.

About breast cancer, in a review article, Cocco et al. reviewed known and emerging prognostic and predictive biomarkers in triple-negative breast cancer (TNBC). This breast cancer subtype is very heterogeneous and is characterized by high aggressivity, distant recurrence risk, and poor survival. The authors summarized the main known genetic (i.e., BRCA1/2) and protein biomarkers used for TNBC prognostic evaluation as well as for their potential to stratify patients for response to the growing number of novel targeted or immunotherapy drugs available to treat this disease. In a research article, Masuda et al. hypothesized that it is possible to predict new breast cancer metastasis (NM) and to guide the treatment of recurrent breast cancer patients by monitoring tumor mesenchymal status in real-time using liquid biopsy. In detail, the authors demonstrated that: (i) N-cadherin and vimentin expression levels were higher in the NM cases compared withpre-existing metastasis cases; (ii) N-cadherin was expressed mainly in polymorphonuclear leukocytes in peripheral blood; and (iii) the preoperative expression levels of N-cadherin were high in breast cancer blood and tissues and associated in a significant statistically way with poor recurrence-free survival. Therefore, N-cadherin mRNA levels in blood were suggested as new putative prognostic biomarkers capable ofpredicting new metastases, including recurrence, in breast cancer patients.

In a review article, Mottini and Cardone focused on the Oncogenic v-Ki-ras2 Kirsten rat sarcoma viral oncogene homolog (KRAS) dependency of pancreatic cancer beyond the mutational status of this oncogene. Indeed, KRAS mutation in pancreatic ductal adenocarcinoma (PDAC) represents a common genetic event that ismutated in almost 88% of cases; however, mutational status is not sufficient to determine whether the tumor is dependent on KRAS and thus is potentially responsive to the novel KRAS inhibitors recently entered into the clinic [11]. Therefore, the authors described the state of KRAS dependency on the basis of transcriptomic and metabolomic profiling studies, highlighting potential molecular biomarkers driven by KRAS mutations in the different PDAC subtypes for a tailored therapy.

Finally, in a research paper, Troiano et al. investigated, in oral squamous cell carcinomas (OSCC), the expression of Musashi 2 (MSI2), a RNA-binding protein that is involved in migration, invasion, epithelial–mesenchymal transition, cell proliferation, and drug resistanceby bioinformatic analysis and immunohistochemical evaluation. TCGA data analysis of 241 OSCC patients showed that the MSI2 gene was not mutated but was rather hypermethylated in all samples analyzed, with higher methylation status correlating with the age of patients and the lowexpression of MSI2 mRNA. Conversely MIS2 mRNA expression levels, being higher in males, correlated with overall survival and grading. Interestingly, although the immunohistochemical evaluation conducted on 108 tissues showeda weak expression of this protein in both OSCC samples and in healthy oral mucosae, the authors highlighted that MSI2 correlated with Cyclin-D1 expression, suggesting an indirect role of MSI2 in OSCC genesis and progression.

In conclusion, although several novel potential biomarkers have been proposed, several authors underlined that robust, well-validated biomarkers are crucial to enabling effective decision-making. Therefore, it is critical to increase the quality and the standardization of the methodology in the development pipeline to select validated and useful prognostic/predictive biomarkers [1].
